# Uteroplacental bleeding disorders during pregnancy: do missing paternal characteristics influence risk?

**DOI:** 10.1186/1471-2393-6-2

**Published:** 2006-02-10

**Authors:** Darios Getahun, Cande V Ananth, Anthony M Vintzileos

**Affiliations:** 1 Division of Epidemiology and Biostatistics, Department of Obstetrics, Gynecology, and Reproductive Sciences, UMDNJ-Robert Wood Johnson Medical School, 125 Paterson Street, New Brunswick, NJ, USA; 2 Division of Maternal-Fetal Medicine, Department of Obstetrics, Gynecology, and Reproductive Sciences, UMDNJ-Robert Wood Johnson Medical School, 125 Paterson Street, New Brunswick, NJ, USA

## Abstract

**Background:**

Several studies have assessed the risks of uteroplacental bleeding disorders in relation to maternal characteristics. The association between uteroplacental bleeding disorders and paternal characteristics, however, has received considerably less attention. Data on paternal demographics, notably race and age, from birth certificate data are becoming increasingly incomplete in recent years. This pattern of increasingly underreporting of paternal demographic data led us to speculate that pregnancies for which paternal characteristics are partially or completely missing may be associated with increased risk for uteroplacental bleeding disorders. The objective of this study is to examine the association between placenta previa and placental abruption and missing paternal age and race.

**Methods:**

A retrospective cohort study using U.S. linked birth/infant death data from 1995 through 2001 (n = 26,336,549) was performed. Risks of placenta previa and placental abruption among: (i) pregnancies with complete paternal age and race data; (ii) paternal age only missing; (iii) paternal race only missing; and (iv) both paternal age and race missing, were evaluated. Relative risk (RR) with 95% confidence interval (CI) for placenta previa and placental abruption by missing paternal characteristics were derived after adjusting for confounders.

**Results:**

Adjusted RR for placental abruption were 1.30 (95% CI 1.24, 1.37), 1.00 (95% CI 0.95, 1.05), and 1.08 (95% CI 1.06, 1.10) among pregnancies with "paternal age only", "paternal race only", and "both paternal age and race" missing, respectively. The increased risk of placental abruption among the "paternal age only missing" category is partly explained by increased risks among whites aged 20–29 years, and among blacks aged ≥30 years. However, no clear patterns in the associations between missing paternal characteristics and placenta previa were evident.

**Conclusion:**

Missing paternal characteristics are associated with increased risk of placental abruption, likely mediated through low socio-economic conditions.

## Background

Placenta previa and placental abruption are the most common uteroplacental bleeding disorders, and are associated with high rate of morbidity and mortality to the mother and fetus[1,2]. In the United States, the prevalence of placenta previa and placental abruption are approximately 3–5 per 1,000 pregnancies, and 5–20 per 1,000 pregnancies respectively[2,3]. The etiologies of placenta previa and placental abruption remain speculative although a variety of socioeconomic factors including maternal race, education, and marital status as well as behavioral and environmental factors have been associated with the occurrence of these conditions. [4-8] In addition, several studies have also reported increased risks of uteroplacental bleeding disorders in relation to various maternal medical and obstetrical complications[2,4,5,9-11]. Previous studies have examined the contributions of paternal characteristics on adverse perinatal outcomes, including paternal age and race. [12-14] The association between uteroplacental bleeding disorders and paternal characteristics, however, has received considerably less attention. The discovery that paternal genes are essential for the normal development and functioning of placenta[15] lends well to the speculation that emphasis on paternal characteristics may play an important role in the genesis of placenta previa and placental abruption. [15-17]

Data on paternal demographics, notably race and age, from birth certificate data are becoming increasingly incomplete in recent years.[18] According to a report of the National Vital Statistics of the United States for the year 2000, paternal age was missing in 14% of the birth certificates,[19] and paternal race missing in 14% of the birth certificates.[20] This pattern of increasingly underreporting of paternal demographic data led us to speculate that (i) pregnancies for which paternal characteristics is partially or completely missing may be associated with poor socio-economic conditions; and (ii) such pregnancies with missing paternal characteristics may be associated with increased risk for uteroplacental bleeding disorders. We recently reported the results of a study on the associations of maternal and paternal race on uteroplacental bleeding disorders.[21] We herein report the associations between missing paternal age and paternal race and risk of uteroplacental bleeding disorders among singleton pregnancies in the United States.

## Methods

### Data source

This analysis used the linked birth/infant death data files from 1995 through 2001, available from the National Center for Health Statistics (NCHS).[22] NCHS routinely links birth certificate and infant death certificate files that are provided by individual State under the Vital Statistics Cooperative Program.[22] Gestational age is defined as the period from the first day of the last normal menstrual period (LMP) to the day of birth. When information on the LMP was unavailable, or when the calculated gestational age was inconsistent with birthweight, the clinical estimate of gestational age, also contained on the vital records, was used.[23,24] For those births with available information on month and year for the last menstrual period but with missing day, the NCHS performed a statistical imputation of gestational age.[23,24]

The study comprised of singleton births delivered at ≥20 weeks of gestation, and fetuses that weighed ≥500 g at birth that occurred to women aged 14 to 45 years. Four non-overlapping cohorts were constructed: i) pregnancies with complete paternal age and paternal race data; ii) pregnancies with paternal age but not race missing; iii) pregnancies with paternal race but not paternal age missing; and iv) pregnancies with both paternal age and paternal race missing.

Variables considered as potential confounders included maternal age (categorized as <20, 20–24, 25–29, 30–34, and ≥35 years), maternal education (categorized as <12, 12, 13–15, and ≥16 years of completed schooling), marital status (married or unmarried), parity (primiparous or multiparous), prenatal care (care initiated in the 1^st ^trimester, 2^nd ^trimester, 3^rd ^trimester, or no prenatal care), smoking during pregnancy (yes or no), and chronic hypertension (yes or no). Self-reported maternal and paternal race were grouped as white and black, irrespective of Hispanic origin. Thus, white race group includes White non-Hispanic and White Hispanic. Similarly, black race group includes Black non-Hispanic and Black Hispanic women. We excluded "other races" owing to relatively small yearly counts. The main outcomes examined in this study were placenta previa (implantation of placenta over or near the internal os of the cervix) and placental abruption (the premature separation of a normally implanted placenta from its implantation in the uterus).[18]

### Data exclusions

We excluded births at <20 completed weeks gestation and fetuses that weighed <500 g from the analysis (n = 408,631). Limiting the analysis to births at ≥20 completed weeks gestation and birthweight ≥500 g was to avoid errors in gestational age and to minimize interstate variations in reporting of live births at borderline viability.[25] Pregnancies to women aged <14 years or >45 years (n = 22,716) and "others" paternal race (n = 1,208,128) also were excluded due to sparse data. After all exclusions were made, 26,336,549 singleton births remained for analyses.

### Statistical analysis

A population-based, retrospective cohort study was performed. To compare the distribution of maternal characteristics at every group of paternal characteristics, we analyzed their frequencies. We computed the distribution of gestational age-specific cumulative percent of placental abruption and placenta previa for each paternal characteristics category. Subsequently, we used multivariable logistic regression analysis to evaluate the association between the outcome variables and the four categories of missing parental characteristics, after adjusting for confounding variables. Adjustments were made for maternal age, maternal education, maternal race, marital status, parity, prenatal care, maternal smoking during pregnancy, chronic hypertension, and prior cesarean delivery. The reference group for the paternal characteristics categories consisted of pregnancies with complete paternal race and age data. Relative risk (RR) and 95% confidence interval (CI) were used to describe the associations.

The study was approved by the Ethics Committee of the Institutional Review Board of UMDNJ-Robert Wood Johnson Medical School, New Brunswick, NJ. All statistical analyses were performed using the SAS software version 9.1 (SAS institute, Cary, NC).

## Results

We identified 4,115,214 (15.6% of the total) records for which data on paternal characteristics was incomplete. This comprised 1.2% (n = 311,938) records with missing paternal race, but not age, 0.8% (n = 212,474) records with missing paternal age, but not race, and 13.6% (n = 3,590,802) records with both paternal age and race missing. Pregnancies with missing data on the father were most likely to occur among women <25 years of age, <12 years of completed education, unmarried, primipara, that had smoked during pregnancy, and initiated prenatal care late during pregnancy (Table 1).

**Table 1:** Maternal characteristic among the four paternal categories: United States, 1995–2001

	**Completeness of paternal characteristics**			
	
**Maternal Characteristics**	**Complete (%) n = 22,221,335**	**Age missing (%) n = 212,474**	**Race missing (%) n = 311,938**	**Both missing (%) n = 3,590,802**
Age (years)				
<20	9.3	23.8	17.9	31.6
20–24	23.2	31.0	28.9	35.3
25–29	28.8	21.5	23.7	17.7
30–34	25.0	14.4	18.1	9.5
≥35	13.7	9.2	11.4	5.9
Education				
<12	18.3	47.0	31.7	44.1
12	31.9	34.2	36.3	38.4
13–15	23.4	13.6	18.7	14.3
≥16	26.5	5.2	13.3	3.2
Race				
White	83.9	70.3	77.8	54.0
Black	11.0	25.0	17.1	42.2
Others	5.1	4.8	5.2	3.8
Prenatal care began				
1st trimester	84.0	63.0	71.6	61.7
2nd trimester	11.2	22.6	16.1	24.1
3rd trimester	2.1	6.2	3.6	6.4
No care	0.7	3.3	1.4	3.8
Missing	2.0	4.9	7.3	4.0
Primiparity	32.3	36.7	37.9	40.7
Unmarried status	22.1	73.1	55.5	96.5
Smoking				
Yes	9.2	16.3	14.2	19.8
No	72.6	48.9	70.5	71.3
Missing	18.2	34.8	15.2	8.9

All comparisons were statistically significant (P < 0.001)

**Figure 1:**
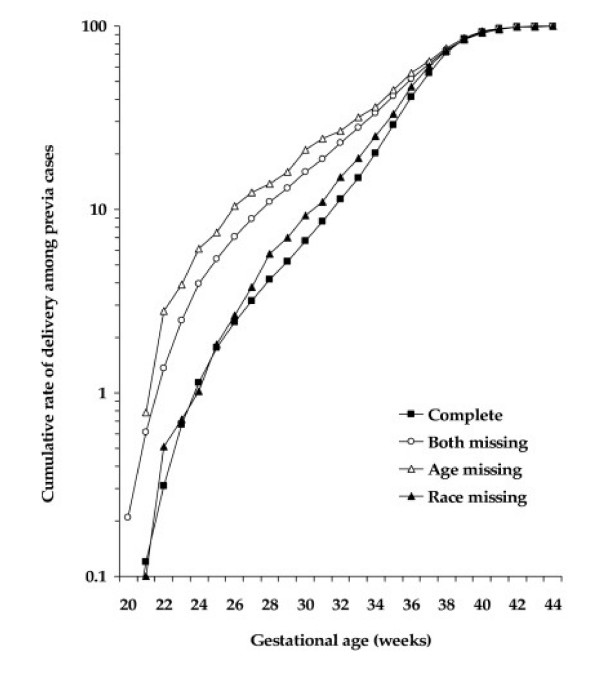
Gestational age-specific cumulative rate of placenta previa by missing paternal characteristics.

**Figure 2:**
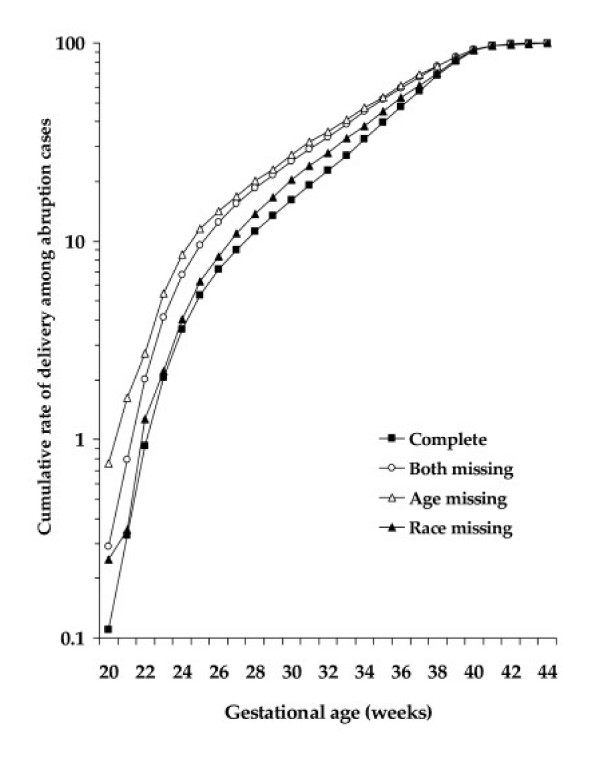
Gestational age-specific cumulative rate of placental abruption by missing paternal characteristics.

Figure 1 and 2 shows gestational age-specific cumulative rates of placenta previa and placental abruption by missing paternal characteristics. The rates of placenta previa and placental abruption in "age only missing" pregnancies were consistently higher at every preterm gestational age indicating higher preterm birth rates compared to complete paternal age and race.

Table 2 shows the crude and adjusted RR for the association between placenta previa and placental abruption and the various missing paternal characteristics. The rate of placenta previa was marginally higher in pregnancies where both paternal age and race were missing, compared with pregnancies where neither paternal age nor race was missing. The relative risk for placental abruption was 1.30 (95% CI 1.24, 1.37) among pregnancies where paternal age only was missing, 1.00 (95% CI 0.95, 1.05) where paternal race only was missing, and 1.08 (95% CI 1.06, 1.10) where both paternal age and race were missing.

**Table 2:** Association uteroplacental bleeding disorders with paternal characteristics: United States, 1995–2001

**Paternal characteristics**	**Rate (%)**	**Relative risk (95% confidence interval)**	
		
		**Unadjusted**	**Adjusted**†
		**Placenta previa**	
Complete	0.33	1.00 (Reference)	1.00 (Reference)
Age only missing	0.30	0.92 (0.85, 1.00)	1.08 (0.99, 1.18)
Race only missing	0.32	0.98 (0.92, 1.04)	1.03 (0.96, 1.11)
Both missing	0.27	0.83 (0.81, 0.85)	1.03 (1.01, 1.06)
		**Placental abruption**	
Complete	0.56	1.00 (Reference)	1.00 (Reference)
Age only missing	1.00	1.80 (1.72, 1.88)	1.30 (1.24, 1.37)
Race only missing	0.65	1.15 (1.10, 1.20)	1.00 (0.95, 1.05)
Both missing	0.80	1.43 (1.41, 1.45)	1.08 (1.06, 1.10)

† Relative risks were adjusted for maternal age, maternal race, parity, maternal education, prenatal care began, marital status, smoking during pregnancy, chronic hypertension, and prior cesarean delivery.

Table 3 shows maternal race- and age-specific adjusted RR for each category of paternal characteristics. The higher rate of placental abruption noted in pregnancies with missing paternal age (RR 1.30) is partly explained by the increased placental abruption rate to white women between 20–29 years, and to black women at ≥30 years. In contrast, the higher rate of placental abruption noted in pregnancies with both age and race were missing is partly explained by the increased rate to white women at ≥20 years. In "both missing" category for blacks, the adjusted RR for placental abruption showed no difference in rates across maternal age strata. The association between paternal characteristics and maternal age-specific rate of placenta previa in pregnancies of both whites and blacks however showed no distinct pattern (Table 4).

**Table 3:** Adjusted relative risk (95% confidence interval) for placental abruption by missing paternal age and race, stratified by maternal race and age

**Paternal characteristics**	**Maternal age (years)**				
	
	**<20**	**20–24**	**25–29**	**30–34**	**≥35**
	**White women**				
Age missing	1.21 (1.05, 1.38)	1.47 (1.32, 1.64)	1.64 (1.45, 1.84)	1.35 (1.16, 1.57)	1.34 (1.13, 1.61)
Race missing	0.98 (0.86, 1.12)	0.99 (0.89, 1.10)	1.05 (0.93, 1.17)	1.04 (0.92, 1.18)	1.13 (0.98, 1.29)
Both missing	1.03 (0.98, 1.07)	1.13 (1.09, 1.18)	1.17 (1.12, 1.23)	1.13 (1.07, 1.21)	1.20 (1.12, 1.29)
	**Black women**				
Age missing	1.31 (1.08, 1.59)	1.21 (1.01, 1.45)	1.35 (1.12, 1.63)	1.77 (1.48, 2.13)	1.50 (1.19, 1.89)
Race missing	1.00 (0.78, 1.27)	1.15 (0.95, 1.38)	0.92 (0.73, 1.16)	0.83 (0.64, 1.09)	0.90 (0.66, 1.22)
Both missing	1.07 (1.01, 1.13)	1.11 (1.06, 1.17)	1.10 (1.04, 1.17)	1.09 (1.00, 1.16)	1.04 (0.95, 1.14)

RRs were adjusted for parity, maternal education, prenatal care, marital status, smoking during pregnancy, chronic hypertension, and prior cesarean delivery and were derived in comparison to "Complete" as the reference group.

**Table 4:** Adjusted relative risk (95% confidence interval) for placenta previa by missing paternal age and race, stratified by maternal race and age

**Paternal characteristics**	**Maternal age (years)**				
	
	**<20**	**20–24**	**25–29**	**30–34**	**≥35**
	**White women**				
Age missing	0.79 (0.54, 1.16)	1.39 (1.14, 1.68)	1.01 (0.82, 1.24)	1.01 (0.82, 1.24)	1.12 (0.91, 1.38)
Race missing	1.02 (0.76, 1.37)	1.27 (1.08, 1.49)	0.98 (0.83, 1.15)	0.95 (0.81, 1.11)	1.18 (1.02, 1.36)
Both missing	1.08 (0.98, 1.19)	1.10 (1.03, 1.18)	1.10 (1.03, 1.18)	1.09 (1.01, 1.17)	1.06 (0.97, 1.14)
	**Black women**				
Age missing	1.27 (0.79, 2.04)	1.11 (0.79, 1.57)	0.91 (0.64, 1.28)	0.98 (0.71, 1.37)	0.93 (0.66, 1.31)
Race missing	0.77 (0.39, 1.48)	0.82 (0.56, 1.21)	0.86 (0.61, 1.22)	1.25 (0.94, 1.68)	1.34 (0.99, 1.78)
Both missing	1.02 (0.89, 1.17)	0.95 (0.87, 1.04)	0.94 (0.86, 1.03)	0.99 (0.89, 1.10)	0.97 (0.87, 1.09)

RRs were adjusted for parity, maternal education, prenatal care, marital status, smoking during pregnancy, chronic hypertension, and prior cesarean delivery and were derived in comparison to "Complete" as the reference group.

To ascertain whether the pattern of missing paternal characteristics is associated with poor social conditions, we examined the association between missing paternal characteristics and placenta previa and placental abruption stratified by marital status, and maternal smoking.

Among unmarried white mothers aged 20–24 years, the RRs for placenta previa were 1.39 (95% CI 1.11, 1.74) when paternal age only was missing, 1.35 (95% CI 1.11, 1.64) when paternal race only was missing, and 1.10 (95% CI 1.03, 1.19) when both age and race were missing. However, among married white mothers aged 20–24 years, these risks were not increased. Similar patterns were evident for analyses pertaining to other maternal age categories. Among black smokers aged <20 years, there was an increased risk of placenta previa when paternal age only was missing (RR 4.48, 95% CI 1.64, 12.24), race only was missing (RR 2.36, 95% CI 0.54, 10.25), and when both race and age were missing (RR 1.27, 95% CI 0.71, 2.26). Among non-smoking black women these risks were not increased. Similar analysis for placental abruption births revealed patterns that were less consistent than those seen for placenta previa.

## Discussion

Several studies have reported associations between maternal demographic and behavioral characteristics and uteroplacental bleeding disorders. The common factors that have been examined include: maternal race, age, marital status, parity, prenatal care, education, cocaine use and smoking during pregnancy. [6,9,26] Studies on the association of paternal characteristics and adverse pregnancy outcomes are, however, scarce. Increased risks of an array of pregnancy outcomes including fetal deaths, preterm delivery, and small-for-gestational age births in relation to paternal age differences,[12] and significantly increased risk of placental abruption among twin pregnancies with missing information on the father have been reported.[27] In this large population based study, we found that missing paternal age and race data was associated with increased risks of uteroplacental bleeding disorders. These associations, albeit limited to placental abruption, were more pronounced in pregnancies where data on paternal age (but not race) was missing.

Pregnancies with missing paternal data were more likely to occur among blacks, unmarried, primiparous, who were <25 years, <12 years of schooling, late initiation of prenatal care, and smoking during pregnancy in comparison to pregnancies with complete information on the father. We hypothesized that pregnancies with missing paternal characteristics would be at increased risk of uteroplacental disorders. This has been partly explained by the higher risk of placental abruption among pregnancies with "age only missing" and "both paternal age and race missing" characteristics as compared with "complete" paternal characteristics. Missing paternal race was not associated with placental abruption. Therefore, we believe that missing paternal age is important determinant of uteroplacental bleeding disorders than missing paternal race. The combined effect of missing paternal age and race (i.e. "both missing") on uteroplacental bleeding disorder is weaker than the effect of "age only missing" and the effect for the latter remained consistently higher at every gestational age (Figure 1). Tan et al.[27] reported that the association between missing paternal demographic and placental abruption among twin births to be significantly higher in pregnancies with paternal information totally missing (RR 1.44, 95% CI 1.27, 1.61), compared with paternal information partially missing (RR 1.03, 95% CI 0.67, 1.39). But, unlike the present study, they failed to show the independent effect of paternal age and race and our data also show that the increased risk of placental abruption and placenta previa in "age only missing" remained consistently higher regardless of gestational age at delivery. Another possible explanation for the higher rate of these uteroplacental bleeding disorders in pregnancies with partial or complete missing paternal information is the higher proportion of pregnancies to women <25 years, <12 years of completed education, unmarried, smoked during pregnancy, primipara, and initiated prenatal care late during pregnancy that are known risk factors for adverse pregnancy outcomes. All these factors point toward the association between missing paternal characteristics and uteroplacental bleeding disorders being largely confined to socioeconomically-disadvantaged women.

Few limitations of the study merit attention. Foremost among them is that the data files are prone to some degree of under-reporting of placental complications, and is likely to contain some coding errors that may introduce random or systemic errors. Second, in spite of our best efforts to adjust for several confounding factors, the possibility of some residual confounding due to unmeasured factors (such as cocaine use, or prepregnancy body-mass index) remain. Finally, data on paternal race is based on either self-report or that reported by the mother. The extent to which such data on vital statistics database are misclassified remains unknown. If missing data on paternal characteristics was due to errors in coding, then these are unlikely to impact maternal outcomes. Further examining the data after stratifying by marital status revealed that the missing paternal information in the vital statistics data may, in fact, not be due to errors in coding alone, but due to other causes. Notwithstanding these shortcomings, this is the largest and most comprehensive population-based study that may have public health implications.

## Conclusion

Missing paternal age appears to be strong determinant for placental abruption, but not for placenta previa. It is likely that such pregnancies occur to socio-economically disadvantaged women.

## List of abbreviations used

University of Medicine and Dentistry of New Jersey (UMDNJ); National Center for Health Statistics (NCHS); Last menstrual period (LMP); Small-for-gestational age (SGA); Relative risk (RR); Confidence interval (CI)

## Competing interests

The author(s) declare that they have no competing interests.

## Authors' contributions

Drs. Getahun and Ananth contributed to the conception and design of the study. Dr. Getahun assembled and analyzed the data, and drafted the manuscript. Drs. Ananth and Vintzileos reviewed the analysis, and edited the manuscript to its final form.

## Pre-publication history

The pre-publication history for this paper can be accessed here:


